# Target‐Specific Dermatological Adverse Event Reporting With T‐Cell–Engaging Bispecific Antibodies: A Pharmacovigilance Analysis of FAERS and Canada Vigilance, 2016–2026

**DOI:** 10.1002/cam4.72241

**Published:** 2026-08-30

**Authors:** Saikat Mandal, Manideepa Maji, Arkadeep Dhali

**Affiliations:** ^1^ Translational Medical Sciences, School of Medicine University of Nottingham Nottingham UK; ^2^ NIHR Nottingham Biomedical Research Centre Nottingham University Hospitals NHS Trust Nottingham UK; ^3^ Hull York Medical School University of Hull Hull UK; ^4^ Haematology Hull University Teaching Hospitals NHS Trust Hull UK; ^5^ Sheffield Teaching Hospitals NHS Trust Sheffield UK; ^6^ School of Medicine, Dentistry and Biomedical Sciences Queen's University Belfast Belfast UK; ^7^ Faculty of Health Imperial College London London UK

**Keywords:** bispecific antibodies, Canada Vigilance, dermatological adverse events, FAERS, GPRC5D, pharmacovigilance, severe cutaneous adverse reactions, talquetamab, tarlatamab, T‐cell engagers

## Abstract

**Background:**

T‐cell–engaging bispecific antibodies are increasingly utilised in haematological malignancies and are beginning to be adopted in solid‐tumour treatment protocols. Cytokine release syndrome and neurotoxicity are well recognised, but target‐specific dermatological adverse event reporting patterns remain poorly defined.

**Methods:**

We analysed 12,333,305 deduplicated FAERS reports from 2016Q1 to 2026Q1, including 23,386 reports exposed to ten T‐cell–engaging bispecific antibodies grouped by target: CD19, CD20, BCMA, GPRC5D and DLL3. A disease‐matched haematologic‐oncology comparator cohort contained 1,062,195 reports. Canada Vigilance provided a secondary directional comparison using 755,911 reports from 2016Q1 to 2025Q4 including 662 unique exposed reports. Outcomes were any cutaneous adverse event, broad severe cutaneous adverse reaction, and narrow Stevens‐Johnson syndrome/toxic epidermal necrolysis. Multivariable models adjusted for age, sex, cancer indication, polypharmacy and classical culprit drugs. Cox models assessed target‐specific timing.

**Results:**

Among exposed FAERS reports, 1394 (5.96%) included any cutaneous adverse event, 61 (0.26%) broad severe cutaneous adverse reaction and 9 (0.04%) narrow Stevens‐Johnson syndrome/toxic epidermal necrolysis. After disease matching, no clear class‐level excess of severe cutaneous reaction reporting was observed. Talquetamab had the highest cutaneous reporting proportion (471/1822; 25.8%) and an elevated timing estimate (hazard ratio 1.34; 95% CI, 1.06–1.69), with enrichment for skin, hair, sweat‐gland and rash phenotypes. Tarlatamab showed an exploratory early‐onset pattern (hazard ratio 3.36; 95% CI, 1.68–6.72; median onset, 4 days), based on only eight reports with usable latency data. Canada Vigilance showed a similar direction of reporting for GPRC5D/any cutaneous adverse events.

**Conclusions:**

T‐cell–engaging bispecific antibodies did not show clear class‐level excess in severe cutaneous adverse‐event reporting after disease matching. Talquetamab showed higher reporting of skin changes, hyperhidrosis, hair abnormalities and rash, whereas tarlatamab showed a possible early‐onset cutaneous reporting pattern based on a small number of reports with usable timing data. These findings support target‐ and timing‐aware dermatological monitoring and require prospective confirmation.

## 1 Introduction

T‐cell–engaging bispecific antibodies (BiTEs) redirect cytotoxic T‐cells against tumour‐associated antigens and have become an important therapeutic class in relapsed or refractory haematological malignancies. Blinatumomab established CD19‐directed T‐cell engagement in B‐cell acute lymphoblastic leukaemia, with randomised evidence in relapsed/refractory and measurable residual disease‐negative settings [1, 2]. The class now includes CD20‐directed agents for B‐cell lymphoma [3], BCMA‐directed teclistamab [4] and GPRC5D‐directed talquetamab [5, 6] for multiple myeloma, and DLL3‐directed tarlatamab for previously treated small‐cell lung cancer [7, 8].

The recognised safety profile of T‐cell–engaging bispecific antibodies includes cytokine release syndrome (CRS), neurotoxicity, cytopenias and infections, alongside target‐specific toxicities [4, 7]. Recent pharmacovigilance studies have identified under‐characterised toxicity patterns with T‐cell–engaging bispecific antibodies [9]. Such disproportionality analyses are hypothesis‐generating and should be interpreted and reported according to established pharmacovigilance guidance [10].

Dermatological adverse events (DAEs) are clinically important because they may impair quality of life, mimic life‐threatening drug hypersensitivity, overlap with CRS, and lead to dose delays or treatment interruption. However, their mechanistic relationship to treatment exposure differs across immunotherapy platforms. CAR T‐cell therapy has been associated with severe cutaneous eruptions and vascular cutaneous reactions, as reported using a 14‐category dermatological grouping [11]. Immune checkpoint inhibitors are associated with a broad spectrum of cutaneous toxicities including severe cutaneous adverse reactions (SCARs), and recent FAERS analyses suggest an independent and synergistic association with SJS/TEN in the presence of classical culprit drugs [12, 13].

For BiTEs, systematic target‐level dermatological pharmacovigilance is lacking. This gap is clinically relevant because GPRC5D is expressed not only on myeloma cells but also in keratinised and adnexal tissues, including hair follicles, sweat glands, tongue epithelium and nail‐associated structures, providing a biologically plausible basis for talquetamab‐associated skin, nail, oral and hair effects [5, 6, 14, 15]. Conversely, tarlatamab's early safety profile is characterised by CRS during step‐up dosing, raising the possibility that cutaneous manifestations may be temporally coupled to cytokine‐release physiology rather than sustained on‐target skin toxicity [7, 8]. Spontaneous‐reporting databases cannot estimate incidence, but they can identify uncommon target‐specific reporting patterns and timing patterns that may be under‐captured in registration trials.

We therefore conducted a FAERS pharmacovigilance analysis from 2016 Q1 through 2026 Q1 to determine whether dermatological adverse event reporting with BiTE therapy reflects a class‐level pattern or distinct molecular target‐specific profiles. We assessed three cutaneous severity tiers, including severe cutaneous reactions and narrow SJS/TEN, mapped events across a 14‐category dermatological grouping, explored target‐specific timing patterns, and examined whether reporting patterns were directionally similar in Canada Vigilance.

## 2 Methods

### 2.1 Data Sources and Cohort Definition

We analysed quarterly FAERS data from the first quarter of 2016 to the first quarter of 2026. Duplicate reports were removed using the standard FAERS method [16]. When more than one report referred to the same case, we retained the most recently submitted report; if two reports had the same submission date, we retained the latest version of that report. The final analytic dataset contained 12,333,305 unique reports. FAERS is a publicly available, de‐identified spontaneous reporting system for post‐marketing safety surveillance; institutional review board approval and informed consent were therefore not required [17]. Reporting follows the principles of the READUS‐PV statement for disproportionality analyses using individual case safety reports [10].

For a secondary directional comparison, we analysed the Health Canada Vigilance Adverse Reaction database from 2016 Q1 to 2025 Q4, using the cumulative extract dated 31 December 2025 [18]. Canada Vigilance contains suspected adverse reaction reports submitted to Health Canada. The report‐level data were combined with information on medicines, adverse reactions, indications, products and active ingredients using the unique report identification number.

### 2.2 Exposure and Comparator Definitions

BiTE exposure was identified by drug name and active ingredient matching against a prespecified dictionary of 10 T‐cell–engaging bispecific antibodies grouped by molecular target: CD19 (blinatumomab), CD20 (glofitamab, epcoritamab, mosunetuzumab, odronextamab), BCMA (teclistamab, elranatamab, linvoseltamab), GPRC5D (talquetamab) and DLL3 (tarlatamab). Because FAERS contains international postmarketing reports, products were retained if they were commercially available or regulatory‐listed during the analysis window, irrespective of jurisdiction. The BiTE product dictionary is provided in Table S1.

Strong and weak SJS/TEN culprit drug groups were defined using drug lists reported in prior FAERS SJS/TEN interaction analyses [13]. Strong culprits included lamotrigine, trimethoprim‐sulfamethoxazole, phenytoin, allopurinol and carbamazepine. Weak culprits comprised azithromycin, clarithromycin, erythromycin, ciprofloxacin, levofloxacin, moxifloxacin and acyclovir. The culprit‐drug dictionary is provided in Table S2.

A disease‐matched haematologic‐oncology comparator cohort was constructed from reports containing non‐BiTE drugs commonly used in BiTE‐eligible indications, including anti‐CD20 and anti‐CD38 monoclonal antibodies, antibody‐drug conjugates, Bruton tyrosine kinase inhibitors, PI3K inhibitors, BCL2 inhibitors, proteasome inhibitors, immunomodulatory drugs, hypomethylating agents, FLT3 and IDH inhibitors and CAR‐T products. Broad cytotoxic drugs with substantial non‐cancer use were excluded. The same product and culprit‐drug dictionaries were applied to Canada Vigilance, where possible. The comparator dictionary is provided in Table S3.

### 2.3 Cutaneous Outcome Definitions

Three prespecified cutaneous outcome tiers were operationalised using MedDRA Preferred Terms. The primary outcome was any cutaneous adverse event (SKIN_ANY), defined as any Preferred Term within the Skin and Subcutaneous Tissue Disorders System Organ Class. The secondary outcome was SCAR‐broad, defined using the Esen et al. MedDRA preferred term query, including Stevens‐Johnson syndrome, toxic epidermal necrolysis, SJS–TEN overlaps, erythema multiforme, drug reaction with eosinophilia and systemic symptoms, acute generalised exanthematous pustulosis, bullous and exfoliative dermatitis terms, cutaneous vasculitis, skin and epidermal necrosis and toxic skin eruption [12]. The tertiary outcome was narrow SJS/TEN, comprising Stevens‐Johnson syndrome, toxic epidermal necrolysis and SJS–TEN overlap [13]. The outcome‐tier dictionary is provided in Table S4.

In parallel, reports were classified into 14 dermatological adverse event categories, based on our previous work and adapted from Storgard et al.: bullous dermatoses, eczematous, psoriasiform, rash, severe cutaneous eruptions, toxic eruptions, urticaria/angioedema, hair changes, hyperhidrosis, pruritus, skin lesions, vascular cutaneous, wound/ulcer and other skin changes [11, 19]. The full Preferred Term mapping is provided in Table S5.

### 2.4 Covariates and Missing Data

Covariates included sex (female, male, unknown), age, cancer indication (binary, derived from indication keyword matching), polypharmacy (number of distinct active ingredients per report), and an age‐missing indicator. Age was missing in approximately 18% of FAERS reports and was imputed using sequential hot‐deck imputation with the 10 covariates most correlated with age serving as donor variables, consistent with prior FAERS SJS/TEN work [13].

### 2.5 Statistical Analysis

For each cutaneous outcome tier, we fitted multivariable logistic regression models including BiTE exposure, strong and weak culprit‐drug exposures, BiTE‐by‐culprit interaction terms, natural spline (age, df = 4), sex, cancer indication, polypharmacy and the age‐missing indicator. Multiplicative interaction was assessed using interaction‐term odds ratios. Additive interaction was estimated using the Relative Excess Risk due to Interaction, Attributable Proportion and Synergy Index with delta‐method standard errors [20, 21].

Two complementary Cox models were used when treatment‐start and adverse‐event dates were available. First, a target‐classified Cox model compared the time from the start of primary‐suspect drug exposure to cutaneous AE onset across BiTE molecular targets, using non‐BiTE primary‐suspect drugs as the reference. In the tables and figures, this corresponds to the target‐classified or mechanism‐based HR. Second, a time‐dependent Cox model used interval splitting, allowing BiTE target exposure to vary during follow‐up and reducing immortal‐time bias. Both models adjusted for age using a penalised spline, sex, cancer indication, polypharmacy and age‐missing status, and included only plausible latencies of 1–180 days.

All class‐level analyses were repeated in the disease‐matched haematologic‐oncology comparator cohort. The 14‐category dermatological panel was analysed using the reporting odds ratio (ROR) with 95% confidence intervals, based on the standard log‐ROR normal approximation. This panel was considered exploratory; nominal elevations were defined by a 95% confidence‐interval lower bound above 1 and were interpreted descriptively rather than as multiplicity‐adjusted confirmatory findings. Because Canada Vigilance lacks a reliable per‐drug start date comparable to the FAERS therapy table, latency models could not be repeated in the Canadian dataset. The Canada analysis was treated as a secondary directional comparison and focused on RORs, with adjusted logistic regression used to assess any cutaneous AE in all‐Canada and disease‐matched haematologic‐oncology cohorts. Statistical significance was defined as two‐sided *p* < 0.05 for prespecified primary analyses. Analyses were conducted in R 4.5.1.

## 3 Results

### 3.1 Cohort Characteristics and Outcome Frequencies

The deduplicated FAERS cohort contained 12,333,305 reports, of which 23,386 (0.19%) included exposure to a BiTE product. The disease‐matched haematologic‐oncology comparator cohort comprised 1,062,195 reports: 23,386 BiTE‐exposed reports and 1,038,809 non‐BiTE haematologic‐oncology reports. Blinatumomab was the largest target‐specific exposure group (9057 reports), followed by CD20‐directed agents (6184 reports across four products), BCMA‐directed agents (5152 reports across three products), talquetamab (1822 reports) and tarlatamab (1378 reports) (Table 1).

**TABLE 1:** Cohort and outcome summary.

Characteristic	All FAERS	BiTE‐exposed	Haematologic‐oncology comparator
Total reports (*n*)	12,333,305	23,386	1,062,195
CD19: blinatumomab	—	9057	—
CD20: glofitamab	—	2006	—
CD20: epcoritamab	—	3367	—
CD20: mosunetuzumab	—	788	—
CD20: odronextamab	—	23	—
BCMA: teclistamab	—	3286	—
BCMA: elranatamab	—	1830	—
BCMA: linvoseltamab	—	36	—
GPRC5D: talquetamab	—	1822	—
DLL3: tarlatamab	—	1378	—
Any cutaneous AE (SKIN_ANY)	1,537,959 (12.5%)	1394 (5.96%)	118,138 (11.1%)
SCAR‐broad (Esen query)	54,151 (0.44%)	61 (0.26%)	4165 (0.39%)
SJS/TEN narrow	17,495 (0.14%)	9 (0.04%)	811 (0.08%)
Strong SJS/TEN culprit	314,936 (2.55%)	3246 (13.9%)	82,210 (7.74%)
Weak SJS/TEN culprit	347,107 (2.81%)	3446 (14.7%)	116,389 (10.96%)

*Note:* Percentages are reported proportions, not incidence rates. Per‐product counts can exceed the total number of BiTE‐exposed reports because some reports contain multiple BiTE products.

Abbreviations: AE, adverse event; BiTE, bispecific T‐cell engager; SCAR, severe cutaneous adverse reaction; SJS/TEN, Stevens‐Johnson syndrome/toxic epidermal necrolysis.

Across all FAERS reports, any cutaneous AE was recorded in 1,537,959 reports (12.5%), SCAR‐broad in 54,151 reports (0.44%) and narrow SJS/TEN in 17,495 reports (0.14%). Among BiTE‐exposed reports, the corresponding counts were 1394 (5.96%), 61 (0.26%) and 9 (0.04%). Per‐product crude cutaneous report proportions varied markedly, from 2.5% for glofitamab to 25.8% for talquetamab (Table 2).

**TABLE 2:** Per‐product crude cutaneous report proportions.

Product	Reports, *n*	Any cutaneous AE	SCAR‐broad	SJS/TEN narrow
Blinatumomab (CD19)	9057	335 (3.7%)	15 (0.17%)	3 (0.03%)
Glofitamab (CD20)	2006	50 (2.5%)	2 (0.10%)	2 (0.10%)
Epcoritamab (CD20)	3367	114 (3.4%)	8 (0.24%)	0 (0.00%)
Mosunetuzumab (CD20)	788	79 (10.0%)	2 (0.25%)	0 (0.00%)
Odronextamab (CD20)	23	1 (4.3%)	0 (0.00%)	0 (0.00%)
Teclistamab (BCMA)	3286	186 (5.7%)	16 (0.49%)	2 (0.06%)
Elranatamab (BCMA)	1830	144 (7.9%)	14 (0.77%)	3 (0.16%)
Linvoseltamab (BCMA)	36	6 (16.7%)	0 (0.00%)	0 (0.00%)
Talquetamab (GPRC5D)	1822	471 (25.8%)	5 (0.27%)	1 (0.05%)
Tarlatamab (DLL3)	1378	38 (2.8%)	1 (0.07%)	0 (0.00%)

*Note:* Values are crude report proportions and should not be interpreted as incidence. SCAR‐broad = severe cutaneous adverse reaction per Esen et al. MedDRA query [12].

### 3.2 Class‐Level Association Across Cutaneous Outcome Tiers

**FIGURE 1:**
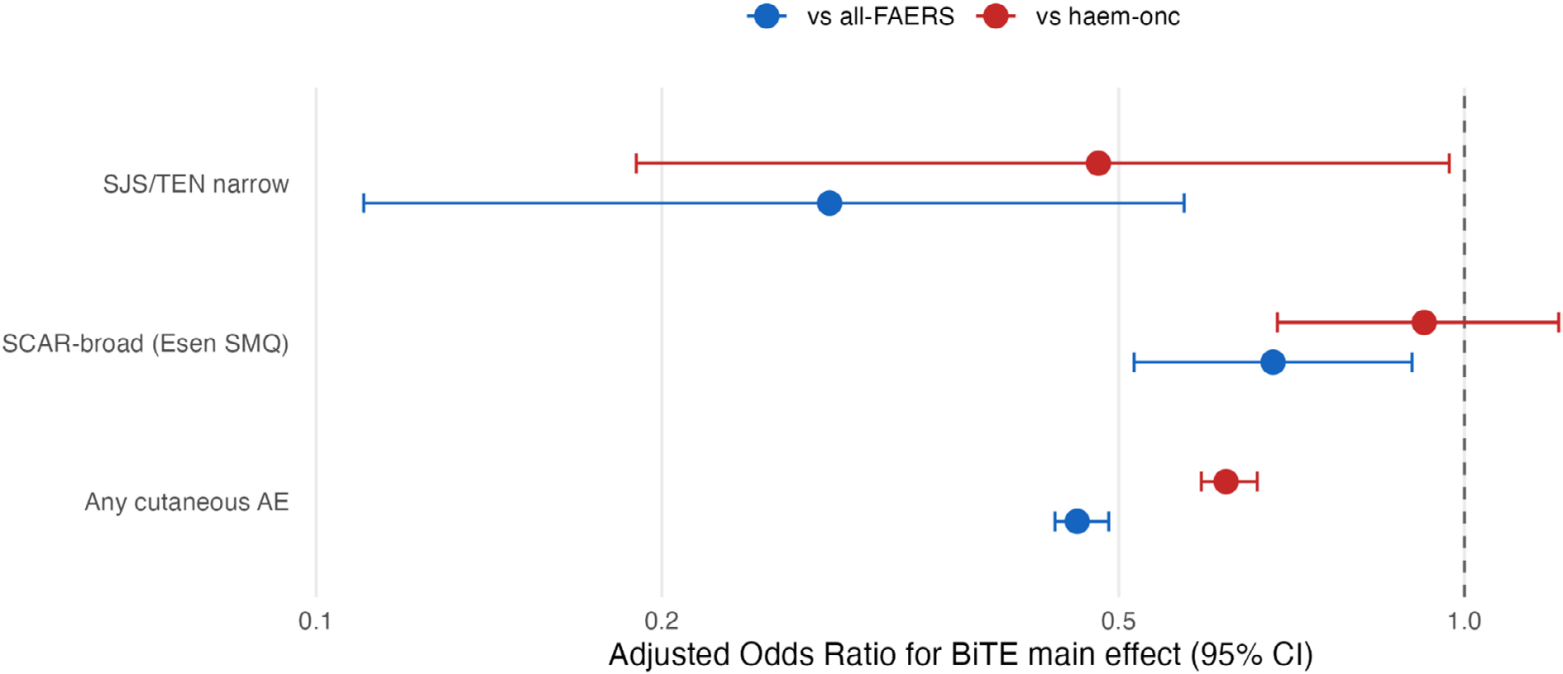
BiTE class‐level association across three cutaneous outcome tiers. Adjusted odds ratios for the BiTE main effect in multivariable logistic regression across any cutaneous adverse event, SCAR‐broad and narrow SJS/TEN, comparing all‐FAERS models with disease‐matched haematologic‐oncology models. The disease‐matched comparator attenuates the apparent inverse reporting association toward the null, particularly for SCAR‐broad.

In all‐FAERS models, BiTE exposure showed inverse reporting associations across each cutaneous outcome tier: adjusted odds ratio (aOR) 0.46 for any cutaneous AE, 0.68 for SCAR‐broad and 0.28 for narrow SJS/TEN. After restriction to the disease‐matched haematologic‐oncology comparator, these estimates attenuated toward the null: 0.62, 0.92 and 0.48, respectively (Figure 1). The SCAR‐broad association (aOR, 0.68; 95% CI, 0.52–0.90; *p* = 0.007) became null in the haematologic‐oncology cohort (aOR, 0.92; 95% CI, 0.69–1.21; *p* = 0.57).

The strong‐culprit drug variable produced the expected dominant signal in the SCAR‐broad model (all‐FAERS aOR: 9.51; 95% CI, 9.28–9.75), supporting the internal consistency of the analysis. The strong‐culprit association attenuated but persisted in the haematologic‐oncology cohort (aOR, 4.06; 95% CI, 3.74–4.42). No super‐additive interaction between BiTE exposure and strong or weak SJS/TEN culprit drugs was detected.

### 3.3 Target‐Specific Cutaneous Reporting Patterns and Timing

**FIGURE 2:**
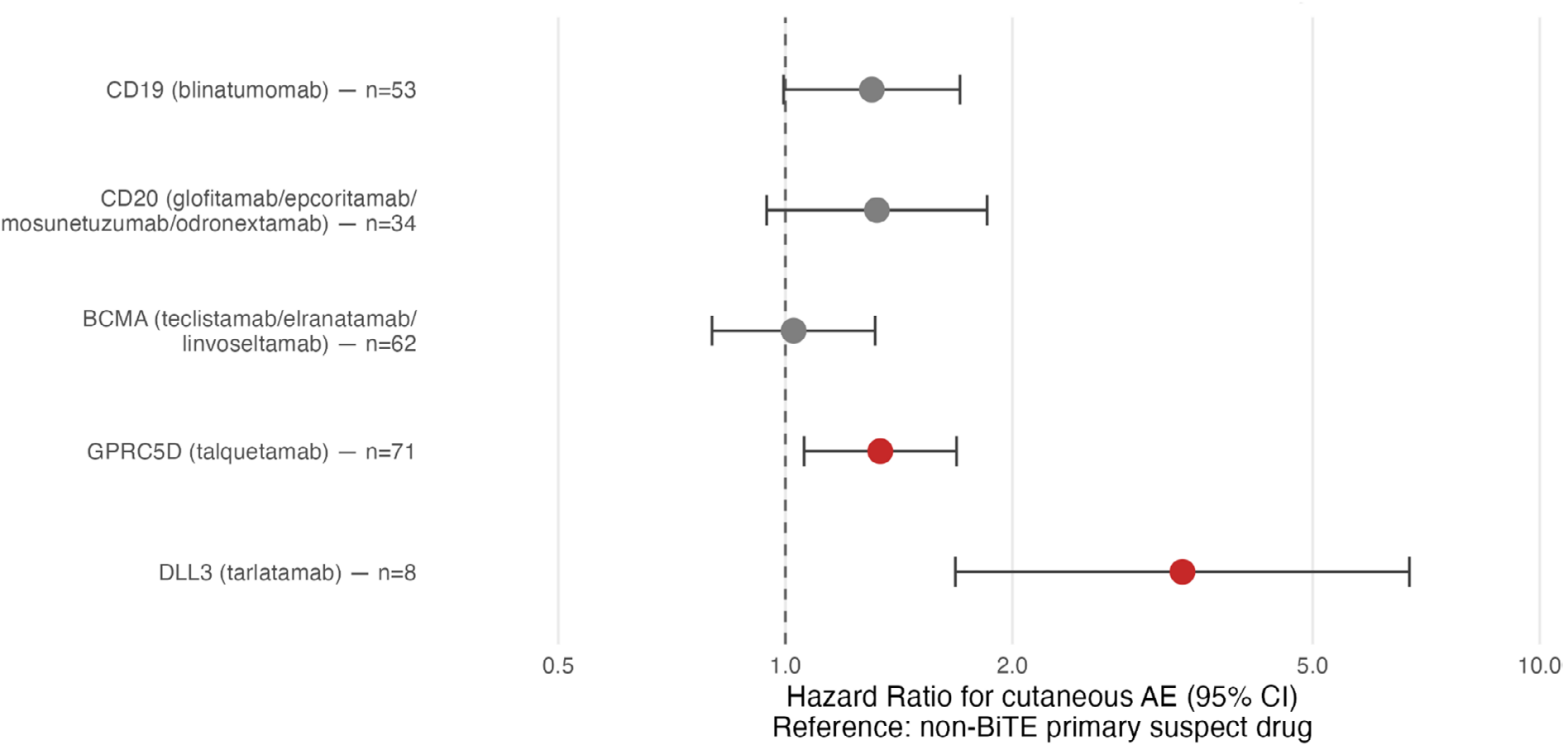
BiTE target‐specific dermatological adverse event timing patterns. Target‐classified Cox proportional hazards estimates with 95% confidence intervals for each BiTE molecular target, with non‐BiTE primary‐suspect drug as the reference. The model was fitted among reports with any cutaneous adverse event and usable latency from the primary‐suspect drug start. Adjustment included age, sex, cancer indication, polypharmacy and age‐missing flag. Estimates based on sparse target‐specific events, particularly DLL3/tarlatamab, should be interpreted as exploratory; red points indicate estimates with 95% confidence intervals excluding 1.

Target‐classified Cox modelling for any cutaneous AE identified two target‐specific timing patterns (Figure 2; Table 3). Talquetamab (GPRC5D) had HR 1.34 (95% CI, 1.06–1.69; *p* = 0.015), based on 71 cutaneous AE cases with usable latency. The time‐dependent Cox model yielded a similar estimate (HR, 1.35; 95% CI, 1.07–1.70; *p* = 0.010). Tarlatamab (DLL3) had a larger timing estimate (target‐classified HR, 3.36; 95% CI, 1.68–6.72; *p* < 0.001), but this was based on only eight reports with usable latency data. The corresponding time‐dependent estimate was similar (HR, 3.43; 95% CI, 1.71–6.85; *p* < 0.001).

**TABLE 3:** Per‐target Cox proportional hazards for any cutaneous adverse event (SKIN_ANY).

BiTE target	Cases (*n*)	Target‐classified Cox HR (95% CI)	Time‐dependent Cox HR (95% CI)	Median onset, day	IQR, day
CD19 (blinatumomab)	53	1.30 (0.99–1.70)	1.39 (1.08–1.77)*	8	2–26
CD20 (4 agents)	34	1.32 (0.94–1.85)	1.19 (0.94–1.52)	15	5–49
BCMA (3 agents)	62	1.03 (0.80–1.32)	1.04 (0.82–1.33)	19	4–53
GPRC5D (talquetamab)	71	1.34 (1.06–1.69)*	1.35 (1.07–1.70)*	15	6–45
DLL3 (tarlatamab)	8	3.36 (1.68–6.72)^†^	3.43 (1.71–6.85)^†^	4	3–7
Non‐BiTE reference	273,022	1.00	1.00	20	6–65

*Note:* Models were fitted among reports with usable latency from primary‐suspect drug start to cutaneous AE onset (1–180 days), adjusted for age, sex, cancer indication, polypharmacy and age‐missing flag. The target‐classified Cox model compares BiTE molecular targets with non‐BiTE primary‐suspect drugs. The DLL3/tarlatamab estimates are based on only eight reports with usable latency and should be interpreted as unstable, descriptive estimates.

Abbreviations: CI, confidence interval; HR, hazard ratio; IQR, interquartile range.

*p < 0.05; ^†^ *p* < 0.001.

**FIGURE 3:**
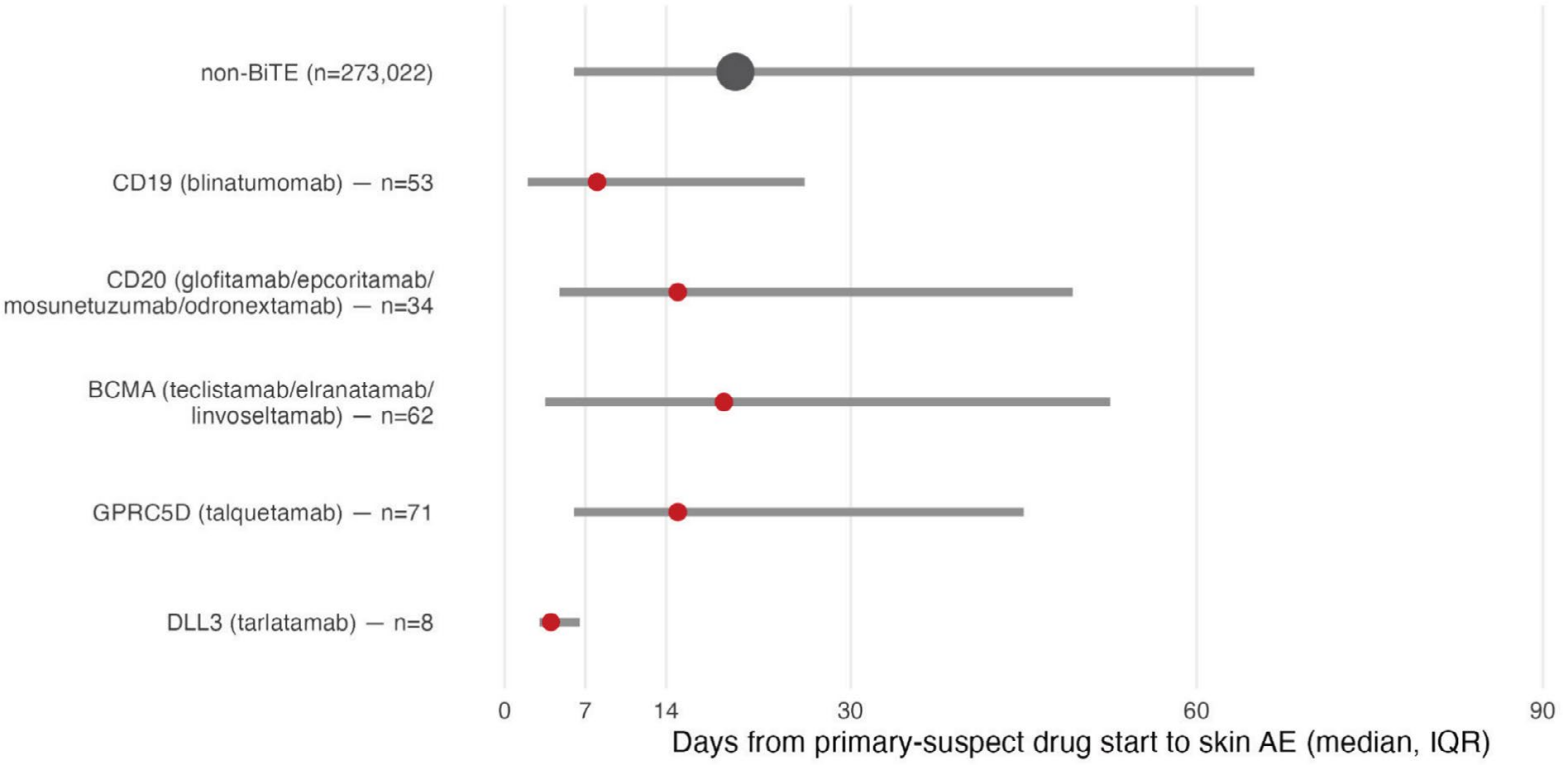
Time to event by BiTE target for any cutaneous adverse event. Median and interquartile range of days from primary‐suspect drug start to cutaneous adverse event onset by BiTE molecular target and non‐BiTE reference, restricted to reports with usable latency. DLL3/tarlatamab reports occurred at a median of 4 days (IQR, 3–7), but this estimate is based on only eight reports and should be considered exploratory. GPRC5D/talquetamab reports occurred at a median of 15 days (IQR, 6–45).

The two patterns differed clinically. Talquetamab had the highest crude cutaneous report proportion (471 of 1822 reports; 25.8%), with median onset of 15 days (IQR, 6–45). Tarlatamab had a lower crude cutaneous report proportion (38 of 1378 reports; 2.8%); among the eight reports with usable latency, onset clustered early, with a median of 4 days (IQR, 3–7) (Figure 3). CD19 and DLL3 reports with usable latency tended to occur earlier, whereas CD20, BCMA and GPRC5D reports were distributed across later onset windows.

Blinatumomab (CD19) showed borderline elevation in the target‐classified model (HR, 1.30; 95% CI, 0.99–1.70) and a statistically significant time‐dependent estimate (HR, 1.39; 95% CI, 1.08–1.77). CD20‐ and BCMA‐directed classes did not show significant elevation in the target‐classified model (CD20 HR, 1.32; 95% CI, 0.94–1.85; BCMA HR, 1.03; 95% CI, 0.80–1.32).

### 3.4 Fourteen‐Category Dermatological Phenotyping

**FIGURE 4:**
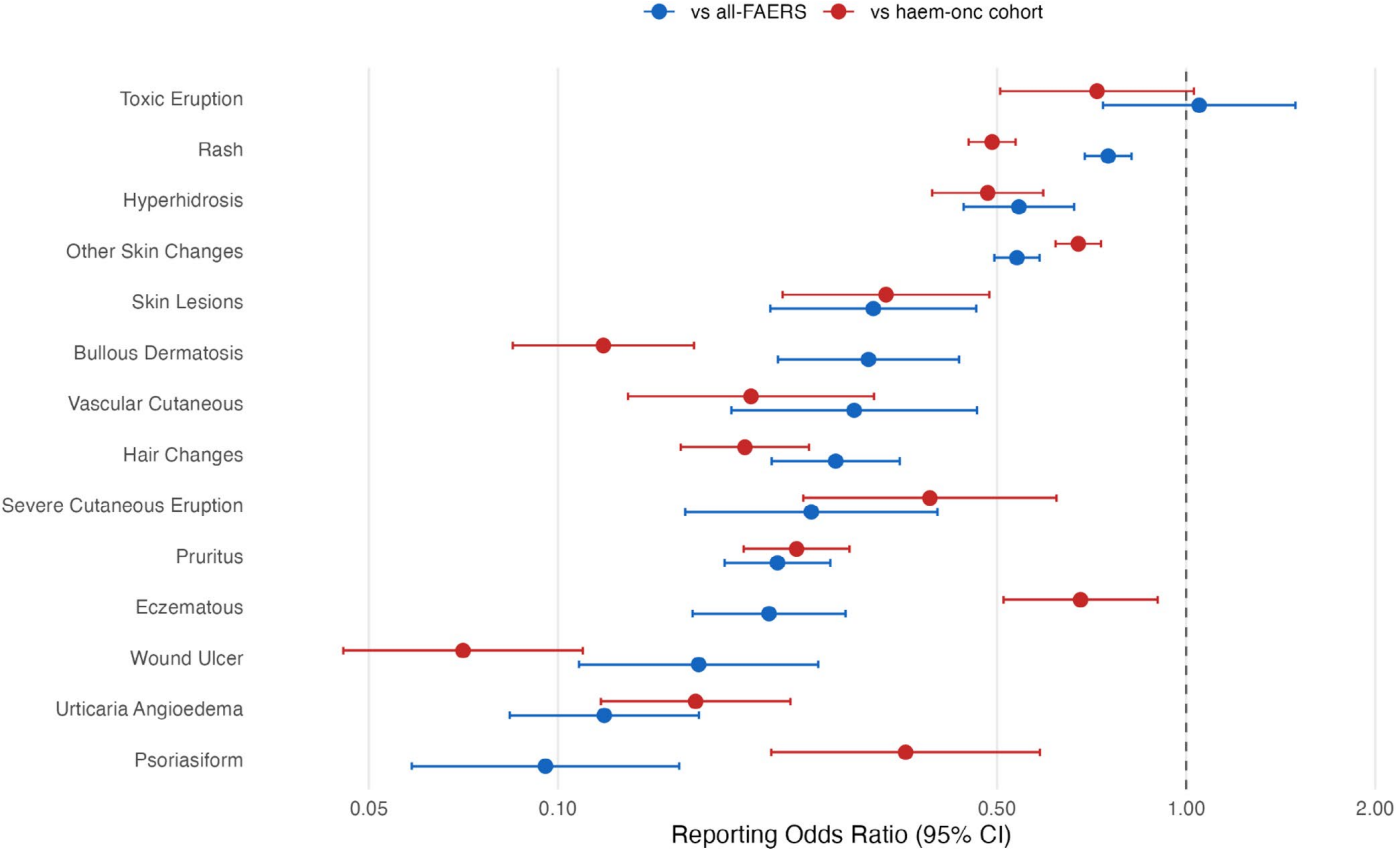
BiTE‐associated cutaneous adverse events across 14 dermatological categories. Reporting odds ratios with 95% confidence intervals for BiTE‐exposed versus non‐BiTE reports across the 14 dermatological adverse event categories, shown for all‐FAERS and disease‐matched haematologic‐oncology comparators. Categories are ordered by all‐FAERS reporting odds ratio.

At the BiTE class level, the 14‐category disproportionality panel showed under‐representation of most dermatological categories relative to all FAERS and haematologic‐oncology comparators (Figure 4).

Per‐target stratification showed the clearest reporting pattern for talquetamab (Table S6). The strongest association was observed for the broad ‘other skin changes’ category (ROR, 5.65; 95% CI, 5.01–6.39), which included erythema, xerosis, skin discolouration, burning or painful skin symptoms, fissuring and other non‐specific cutaneous changes. Talquetamab also showed higher reporting of hyperhidrosis (ROR, 2.03; 95% CI, 1.41–2.92), hair abnormalities (ROR, 1.78; 95% CI, 1.33–2.38) and rash (ROR, 1.49; 95% CI, 1.24–1.80). BCMA‐directed agents showed an isolated toxic‐eruption estimate (ROR, 1.82; 95% CI, 1.13–2.94). Tarlatamab showed no individual category‐level elevation, consistent with the small number of reported cutaneous events.

### 3.5 Canada Vigilance Directional Comparison

The Canada Vigilance cohort contained 662 unique BiTE‐exposed reports within 755,911 reports from 2016 Q1 to 2025 Q4. Product‐ and target‐level counts summed to 663 (367 BCMA, 131 CD19, 81 CD20, 47 DLL3 and 37 GPRC5D) because one report listed more than one BiTE product or target. Narrow SJS/TEN was absent, and SCAR‐broad was represented by one BiTE‐exposed report, precluding meaningful assessment of rare severe cutaneous outcomes in this dataset.

For any cutaneous AE, unadjusted class‐level RORs were directionally similar to FAERS (all‐Canada ROR, 0.61; 95% CI, 0.46–0.81; haematologic‐oncology ROR, 0.57; 95% CI, 0.43–0.75), whereas adjusted estimates moved toward the null (haematologic‐oncology aOR, 1.25; 95% CI, 0.93–1.64). The GPRC5D/talquetamab estimate was directionally higher (haematologic‐oncology ROR, 1.77; 95% CI, 0.81–3.88) and again involved other skin changes. DLL3/tarlatamab did not show a similar directional pattern (ROR, 0.29; 95% CI, 0.07–1.18), with only two tarlatamab cutaneous reports available (Tables S7 and S8 and Figures S1–S3).

## 4 Discussion

### 4.1 Principal Findings

In this pharmacovigilance analysis of 12.3 million FAERS reports, BiTEs did not show a clear class‐level excess in reporting of severe cutaneous adverse reactions after disease matching. The most clinically interpretable findings were target‐specific reporting patterns. Talquetamab showed frequent cutaneous reporting and higher reporting of keratinised and adnexal tissue phenotypes. Tarlatamab showed an uncommon, exploratory pattern of early‐onset cutaneous reporting within the CRS surveillance window, based on only eight reports with usable latency. CD20‐ and BCMA‐directed agents did not show a class‐defining cutaneous timing pattern in the target‐classified Cox analysis. However, BCMA‐directed agents showed an isolated nominal increase in toxic‐eruption reporting (ROR, 1.82; 95% CI, 1.13–2.94), which should be considered exploratory given the absence of a broader BCMA‐associated cutaneous reporting pattern. Blinatumomab showed relatively early cutaneous event reporting, with a median onset of 8 days and an elevated estimate in the time‐dependent Cox model, although the target‐classified estimate did not clearly exclude the null. Dermatological adverse events, particularly rash, have previously been described with blinatumomab, including events occurring early during treatment, although published clinical data suggest considerable variability in time to onset [22].

### 4.2 Talquetamab and Keratinised‐Tissue Biology

Talquetamab's reporting pattern is compatible with its known tissue biology. GPRC5D expression has been documented in plasma cells and in keratinised or adnexal tissues, including hair follicles, eccrine sweat glands, tongue epithelium and nail‐associated structures [5, 6, 14, 15]. In Phases 1 and 2 of the MonumenTAL‐1 trial, talquetamab was associated with common, mostly low‐grade skin‐related events and dysgeusia, supporting the biological plausibility of GPRC5D‐associated on‐target, off‐tumour effects [5]. The present FAERS findings were consistent with this biological rationale. The strongest association involved the broad ‘other skin changes’ category, comprising erythema, xerosis, skin discolouration, burning or painful skin symptoms, fissuring and related cutaneous changes. Higher reporting of hyperhidrosis, hair abnormalities and rash was also observed. However, spontaneous reporting data cannot establish causality or confirm an underlying biological mechanism.

Clinically, this pattern supports awareness of skin, nail, hair, oral and sweat‐gland symptoms during talquetamab treatment, consistent with published practical guidance [23]. Baseline documentation may assist attribution, and patients should be counselled about red flags such as blistering, mucosal involvement, fever, pain, skin detachment or rapidly progressive eruption. However, reporting bias is an important alternative explanation. Talquetamab‐associated dermatological toxicities are already well recognised from clinical trials and regulatory information, so increased clinician and patient awareness may increase reporting of expected events. Tarlatamab is also a relatively new therapy with substantial attention to early treatment toxicities. The observed patterns may therefore reflect both underlying drug effects and differential reporting intensity, and their magnitude should not be interpreted as comparative clinical risk.

### 4.3 Tarlatamab and Early CRS‐Coupled Cutaneous Reports

Tarlatamab's pattern differed from talquetamab's. The crude cutaneous report proportion was low, and the timing estimate was based on only eight reports with usable latency, with a median onset of 4 days. Although the direction of the estimate was similar in both Cox models, the small event count makes the magnitude of the hazard ratio unstable and potentially sensitive to individual reports. The observed early clustering is therefore descriptive and hypothesis‐generating and cannot distinguish CRS‐associated cutaneous manifestations from unrelated skin events. Tarlatamab's label includes boxed warnings for CRS and neurologic toxicity, and the DeLLphi‐301 programme established CRS as a common early toxicity [7, 8].

If confirmed, this early timing pattern would support careful documentation of skin findings during initial and step‐up dosing. Rash, flushing, erythema, urticaria‐like symptoms or nonspecific inflammatory skin findings should be interpreted alongside CRS grade, antipyretic or corticosteroid exposure and concurrent culprit drugs. Current pharmacovigilance data are insufficient to define a specific causal risk window, and suspected SCAR or progressive hypersensitivity should continue to prompt urgent clinical assessment and dermatology input.

### 4.4 Class‐Level Interpretation and Comparison With Immune Checkpoint Inhibitors

The disease‐matched SCAR‐broad result (aOR, 0.92; 95% CI, 0.69–1.21) did not show excess reporting of severe cutaneous events with BiTE exposure compared with clinically relevant haematologic‐oncology comparators. This should not be interpreted as evidence of a lower clinical risk, because spontaneous reporting data do not provide incidence estimates. The pattern contrasts with immune checkpoint inhibitor analyses, in which ICI exposure was associated with SJS/TEN and showed additive synergy with classical culprit drugs [13]. Although both treatment classes activate T‐cells, their reported cutaneous‐event profiles may differ.

The comparison also underscores why a disease‐matched comparator is essential. In all‐FAERS analyses, BiTE exposure showed inverse reporting associations with cutaneous outcomes, but these attenuated after disease matching. This apparent inverse association is best interpreted as a population‐mix and reporting‐profile artefact rather than a protective effect: BiTE reports are enriched for CRS, neurotoxicity, cytopenias, infection and haematologic‐oncology supportive‐care events, which can reduce the relative share of dermatological reports.

### 4.5 Canada Vigilance Directional Comparison

Canada Vigilance provided a secondary directional comparison rather than an independent validation. Only 662 BiTE‐exposed reports were available, with one broad SCAR report and no SJS/TEN reports, so the Canadian dataset cannot confirm severe cutaneous outcomes. The GPRC5D/talquetamab estimate was directionally similar to FAERS for any cutaneous AE and other skin changes, but confidence intervals were wide. DLL3/tarlatamab could not be meaningfully assessed because only 47 tarlatamab reports and two cutaneous events were available. These findings should therefore be regarded as supportive and hypothesis‐generating only.

### 4.6 Strengths and Limitations

Strengths include a large, contemporary FAERS dataset; prespecified three‐tier cutaneous outcome definitions; prespecified strong‐ and weak‐culprit‐drug groups; disease‐matched sensitivity analyses; a secondary Canada Vigilance directional comparison; and complementary target‐classified and time‐dependent Cox models. The analysis also integrates a biologically interpretable 14‐category dermatological panel rather than relying solely on broad system‐organ‐class outcomes.

Several limitations require caution. FAERS and Canada Vigilance are passive spontaneous reporting systems; reported proportions are not incidence rates, and RORs, aORs and HRs describe reporting associations or timing patterns rather than causal risk. Reports may contain missing dates, incomplete co‐medication data, misclassified Preferred Terms, duplicate clinical narratives not fully removed by structured deduplication, and reporting biases driven by drug novelty, label changes, media attention, clinician awareness and known toxicity profiles, including notoriety bias. The tarlatamab timing analysis was based on only eight reports with usable treatment‐start and event‐onset dates. Although the direction of the estimate was similar in both Cox models, the small event count makes the magnitude of the hazard ratio unstable and highly sensitive to individual reports; this finding should therefore be considered descriptive and hypothesis‐generating. Canada Vigilance lacked reliable drug‐start dates, included only one BiTE‐exposed broad SCAR report and no BiTE‐exposed SJS/TEN reports, and was not sufficiently powered to confirm rare severe outcomes. Residual confounding by indication, treatment line, dose, inpatient monitoring intensity, CRS severity, infection and co‐medication cannot be excluded. Prospective registries and electronic health record cohorts with denominator exposure, clinician‐adjudicated skin phenotypes and treatment‐timing detail are needed.

## 5 Conclusions

In contemporary pharmacovigilance data, T‐cell–engaging bispecific antibodies did not show a clear class‐level excess in severe cutaneous adverse‐event reporting after disease matching. Talquetamab was associated with increased reporting of a broad range of cutaneous changes, including erythema, xerosis, skin discolouration and fissuring, together with hyperhidrosis, hair abnormalities and rash; this pattern is consistent with GPRC5D expression in keratinised and adnexal tissues including hair follicles, sweat glands and nail‐associated structures. Tarlatamab showed a possible early‐onset cutaneous reporting pattern, with events occurring within the first few days of treatment; however, this observation was based on very few reports with complete timing information and should be regarded as hypothesis‐generating. These findings support careful dermatological assessment according to the molecular target and timing of therapy, but spontaneous reporting data cannot determine true incidence or establish causality. Prospective studies are needed to confirm these patterns and clarify their clinical significance.

## Author Contributions

**Arkadeep Dhali:** writing – original draft, writing – review and editing, methodology, formal analysis. **Saikat Mandal:** conceptualization, methodology, visualization, writing – original draft, formal analysis, writing – review and editing. **Manideepa Maji:** conceptualization, writing – original draft, writing – review and editing, methodology, formal analysis.

## Funding

S.M. is supported by NIHR Nottingham Biomedical Research Centre [IS‐BRC‐1215‐20003]. Job role of S.M. is funded through UK Research and Innovation grant MR/Z505183/1. Job role of M.M. and A.D. is funded through NIHR Academic Clinical Fellowship.

## Ethics Statement

This study used publicly available, de‐identified FAERS and Canada Vigilance data; ethics approval and informed consent were not required.

## Conflicts of Interest

The authors declare no conflicts of interest.

## Supporting information


**Table S1:** BiTE product and target exposure dictionary.
**Table S2:** Strong and weak SJS/TEN culprit‐drug dictionary.
**Table S3:** Disease‐matched haematologic‐oncology comparator dictionary.
**Table S4:** Cutaneous outcome‐tier dictionary.
**Table S5:** PT‐to‐category mapping.
**Table S6:** Per‐target × category disproportionality.
**Table S7:** Canada Vigilance per‐product report counts and cutaneous outcome counts.
**Table S8:** Canada Vigilance target‐specific reporting odds ratios for any cutaneous adverse event.
**Figure S1:** Canada Vigilance BiTE association across two cutaneous tiers.
**Figure S2:** Canada Vigilance 14‐category cutaneous disproportionality panel.
**Figure S3:** Canada Vigilance target‐specific cutaneous AE reporting pattern.

## Data Availability

FAERS and Canada Vigilance data are publicly available from their respective regulatory portals. The exposure dictionaries and outcome definitions used for cohort construction are provided in Tables S1–S8 accompanying this submission. The analysis scripts, exposure dictionaries, outcome definitions, and Canada Vigilance directional‐comparison outputs will be available from the corresponding author upon reasonable request.
