# Supplementary material for: Target‐Specific Dermatological Adverse Event Reporting With T‐Cell–Engaging Bispecific Antibodies: A Pharmacovigilance Analysis of FAERS and Canada Vigilance, 2016–2026
Source: Cancer Med. 2026 Aug 30;15(9):e72241. doi: 10.1002/cam4.72241 (PMC13527524; doi:10.1002/cam4.72241)
Supplement: Supplementary file 1 [file CAM4-15-e72241-s001.docx]

**Supplementary Material - Target-Specific Dermatological Adverse Event Reporting With T-Cell–Engaging Bispecific Antibodies: A Pharmacovigilance Analysis of FAERS and Canada Vigilance, 2016-2026**

# **Supplementary Materials Contents**

Supplementary Table S1: BiTE product and target exposure dictionary.

Supplementary Table S2: Strong and weak SJS/TEN culprit-drug dictionary.

Supplementary Table S3: Disease-matched haematologic-oncology comparator dictionary.

Supplementary Table S4: Cutaneous outcome-tier dictionary.

Supplementary Table S5: MedDRA Preferred Terms matched to the 14 dermatological adverse event categories.

Supplementary Table S6: Per-BiTE-target × Storgard category disproportionality analysis, restricted to the disease-matched haematologic-oncology comparator cohort.

Supplementary Table S7: Canada Vigilance per-product report counts and cutaneous outcome counts.

Supplementary Table S8: Canada Vigilance target-specific reporting odds ratios for any cutaneous adverse event.

Supplementary Figures S1-S3: Canada Vigilance directional comparison figures.

**Supplementary Table S1: BiTE product and target exposure dictionary**

Products were grouped by molecular target. Matching terms include active ingredient and common brand-name or suffix variants used in pharmacovigilance drug-name fields.

| **Product** | **Target** | **Matching terms** |
| --- | --- | --- |
| Blinatumomab | CD19 | blinatumomab; blincyto |
| Glofitamab | CD20 | glofitamab; columvi |
| Epcoritamab | CD20 | epcoritamab; epkinly; tepkinly |
| Mosunetuzumab | CD20 | mosunetuzumab; lunsumio |
| Odronextamab | CD20 | odronextamab; ordesekimab; odronextamab-cxdl |
| Teclistamab | BCMA | teclistamab; tecvayli |
| Elranatamab | BCMA | elranatamab; elrexfio |
| Linvoseltamab | BCMA | linvoseltamab |
| Talquetamab | GPRC5D | talquetamab; talvey |
| Tarlatamab | DLL3 | tarlatamab; imdelltra; tarlatamab-dlle |

**Supplementary Table S2: Strong and weak SJS/TEN culprit-drug dictionary**

Strong and weak SJS/TEN culprit-drug groupings were based on drug lists reported by Mukherjee et al. and were applied to FAERS and Canada Vigilance where possible.

| **Culprit group** | **Matching terms** |
| --- | --- |
| Strong SJS/TEN culprit | lamotrigine; trimethoprim-sulfamethoxazole; trimethoprim; sulfamethoxazole; phenytoin; allopurinol; carbamazepine |
| Weak SJS/TEN culprit | azithromycin; clarithromycin; erythromycin; ciprofloxacin; levofloxacin; moxifloxacin; acyclovir |

**Supplementary Table S3: Disease-matched haematologic-oncology comparator dictionary**

The comparator cohort comprised reports containing at least one non-BiTE haematologic-oncology product commonly used in BiTE-eligible disease settings. Broad cytotoxic drugs with substantial non-cancer use were excluded.

| **Drug group** | **Representative matching terms** |
| --- | --- |
| Anti-CD20 monoclonal antibodies | rituximab; obinutuzumab; ofatumumab |
| Anti-CD38/SLAMF7 antibodies | daratumumab; isatuximab; elotuzumab |
| Antibody-drug conjugates / targeted antibodies | brentuximab vedotin; polatuzumab vedotin; inotuzumab ozogamicin; gemtuzumab ozogamicin; belantamab mafodotin; loncastuximab tesirine; tafasitamab |
| BTK inhibitors | ibrutinib; acalabrutinib; zanubrutinib; pirtobrutinib |
| PI3K inhibitors | idelalisib; duvelisib; copanlisib; umbralisib |
| BCL2 inhibitor | venetoclax |
| Proteasome inhibitors | bortezomib; carfilzomib; ixazomib |
| Immunomodulatory drugs | thalidomide; lenalidomide; pomalidomide |
| Hypomethylating agents | azacitidine; decitabine |
| FLT3 inhibitors | midostaurin; gilteritinib; quizartinib |
| IDH inhibitors | ivosidenib; enasidenib; olutasidenib |
| JAK/myelofibrosis agents | ruxolitinib; fedratinib; pacritinib; momelotinib |
| BCR-ABL inhibitors | imatinib; dasatinib; nilotinib; bosutinib; ponatinib; asciminib |
| CAR-T products | tisagenlecleucel; axicabtagene ciloleucel; brexucabtagene autoleucel; lisocabtagene maraleucel; idecabtagene vicleucel; ciltacabtagene autoleucel; obecabtagene autoleucel |

**Supplementary Table S4: Cutaneous outcome-tier dictionary**

Three nested cutaneous outcome tiers were used for the primary and sensitivity analyses.

| **Outcome tier** | **Operational definition / terms** |
| --- | --- |
| Any cutaneous AE (SKIN_ANY) | Any MedDRA Preferred Term in the Skin and Subcutaneous Tissue Disorders System Organ Class; implemented in parallel with the Storgard 14-category bindings. |
| SCAR-broad | Broad severe cutaneous adverse reaction query based on the Esen et al. MedDRA PT set: Stevens-Johnson syndrome; Toxic epidermal necrolysis; SJS-TEN overlap; Erythema multiforme; Drug reaction with eosinophilia and systemic symptoms; Acute generalised exanthematous pustulosis; Dermatitis bullous; Dermatitis exfoliative; Dermatitis exfoliative generalised; Exfoliative rash; Cutaneous vasculitis; Skin necrosis; Epidermal necrosis; Toxic skin eruption; Severe cutaneous adverse reaction; Generalised bullous fixed drug eruption; Oculomucocutaneous syndrome; Target skin lesion |
| SJS/TEN narrow | Stevens-Johnson syndrome; toxic epidermal necrolysis; SJS-TEN overlap |

# **Supplementary Table S5: PT-to-category mapping**

Top MedDRA Preferred Terms by reporting frequency within each of the 14 Storgard et al. (Transplantation and Cellular Therapy 2024) dermatological adverse event categories, derived from FAERS 2016Q1-2026Q1 deduplicated reports. The regex-based PT-to-category mapping is presented for transparency and auditability of the category construction process. Counts reflect total reports across the full FAERS cohort (not BiTE-restricted). Obvious non-clinical, procedural or neoplastic artefacts captured by broad regex bindings were removed from this display table before submission.

| **Category** | **MedDRA Preferred Term** | **n reports** |
| --- | --- | --- |
| **Bullous Dermatosis** | Blister | 33,371 |
|  | Pemphigus | 10,739 |
|  | Pemphigoid | 4,815 |
|  | Oral mucosal blistering | 3,800 |
|  | Dermatitis bullous | 3,397 |
|  | Blood blister | 1,618 |
|  | Lip blister | 1,488 |
|  | Tongue blistering | 1,080 |
|  | Linear iga disease | 939 |
|  | Blister rupture | 508 |
|  | Oropharyngeal blistering | 487 |
|  | Blister infected | 454 |
|  | Generalised bullous fixed drug eruption | 263 |
|  | Genital blister | 225 |
| **Eczematous** | Dermatitis atopic | 44,053 |
|  | Eczema | 37,976 |
|  | Dermatitis | 14,076 |
|  | Dermatitis allergic | 7,599 |
|  | Dermatitis contact | 6,633 |
|  | Neurodermatitis | 2,679 |
|  | Rebound eczema | 2,033 |
|  | Rebound atopic dermatitis | 2,019 |
|  | Seborrhoeic dermatitis | 937 |
|  | Immune-mediated dermatitis | 821 |
|  | Acarodermatitis | 646 |
|  | Perioral dermatitis | 555 |
|  | Hand dermatitis | 552 |
|  | Dyshidrotic eczema | 489 |
|  | Eczema eyelids | 437 |
| **Psoriasiform** | Psoriasis | 83,590 |
|  | Pustular psoriasis | 1,688 |
|  | Dermatitis psoriasiform | 1,228 |
|  | Rebound psoriasis | 513 |
|  | Guttate psoriasis | 447 |
|  | Palmoplantar pustulosis | 443 |
|  | Nail psoriasis | 435 |
|  | Erythrodermic psoriasis | 397 |
|  | Paradoxical psoriasis | 356 |
|  | Generalised pustular psoriasis | 203 |
|  | Psoriasis area severity index increased | 136 |
|  | Parapsoriasis | 128 |
|  | Psoriasis area severity index decreased | 11 |
|  | Psoriasis area severity index | 4 |
| **Rash** | Rash | 271,017 |
|  | Rash pruritic | 30,207 |
|  | Rash erythematous | 27,638 |
|  | Rash macular | 21,984 |
|  | Rash papular | 12,858 |
|  | Rash maculo-papular | 11,464 |
|  | Drug eruption | 9,530 |
|  | Toxic skin eruption | 4,465 |
|  | Acute generalised exanthematous pustulosis | 3,794 |
|  | Rash vesicular | 3,007 |
|  | Rash pustular | 2,875 |
|  | Rash morbilliform | 1,425 |
|  | Fixed eruption | 1,259 |
|  | Oral mucosal eruption | 998 |
|  | Symmetrical drug-related intertriginous and flexural exanthema | 607 |
| **Severe Cutaneous Eruption** | Drug reaction with eosinophilia and systemic symptoms | 15,702 |
|  | Stevens-johnson syndrome | 8,224 |
|  | Toxic epidermal necrolysis | 6,957 |
|  | Erythema multiforme | 4,227 |
|  | Acute generalised exanthematous pustulosis | 3,794 |
|  | Sjs-ten overlap | 533 |
|  | Severe cutaneous adverse reaction | 178 |
|  | Purpura fulminans | 161 |
|  | Oculomucocutaneous syndrome | 119 |
|  | Agep-dress overlap | 22 |
|  | Atypical stevens-johnson syndrome | 7 |
| **Toxic Eruption** | Drug eruption | 9,530 |
|  | Toxic skin eruption | 4,465 |
|  | Fixed eruption | 1,259 |
|  | Generalised bullous fixed drug eruption | 263 |
|  | Toxic erythema of chemotherapy | 186 |
|  | Fixed drug eruption | 75 |
| **Urticaria Angioedema** | Urticaria | 97,092 |
|  | Angioedema | 23,182 |
|  | Hereditary angioedema | 18,460 |
|  | Injection site urticaria | 14,118 |
|  | Application site urticaria | 879 |
|  | Chronic spontaneous urticaria | 808 |
|  | Idiopathic urticaria | 738 |
|  | Infusion site urticaria | 524 |
|  | Urticarial vasculitis | 418 |
|  | Urticaria chronic | 352 |
|  | Mechanical urticaria | 304 |
|  | Intestinal angioedema | 292 |
|  | Cold urticaria | 159 |
|  | Urticaria papular | 98 |
|  | Urticaria thermal | 94 |
| **Hair Changes** | Alopecia | 117,139 |
|  | Hair texture abnormal | 5,331 |
|  | Hair colour changes | 5,096 |
|  | Madarosis | 4,818 |
|  | Hair growth abnormal | 3,806 |
|  | Hair disorder | 2,277 |
|  | Trichorrhexis | 1,536 |
|  | Diffuse alopecia | 1,285 |
|  | Alopecia areata | 1,051 |
|  | Hypertrichosis | 940 |
|  | Alopecia universalis | 169 |
|  | Androgenetic alopecia | 151 |
|  | Alopecia totalis | 147 |
|  | Hair growth rate abnormal | 122 |
|  | Alopecia scarring | 111 |
| **Hyperhidrosis** | Hyperhidrosis | 64,991 |
|  | Night sweats | 18,205 |
|  | Cold sweat | 8,669 |
|  | Miliaria | 1,173 |
|  | Hypohidrosis | 257 |
|  | Anhidrosis | 206 |
|  | Sweat discolouration | 78 |
|  | Sweat gland disorder | 62 |
| **Pruritus** | Pruritus | 240,445 |
|  | Pruritus allergic | 736 |
|  | Tumour pruritus | 6 |
| **Skin Lesions** | Skin lesion | 15,654 |
|  | Scab | 7,117 |
|  | Skin plaque | 5,575 |
|  | Papule | 4,922 |
|  | Skin mass | 4,377 |
|  | Injection site papule | 2,126 |
|  | Macule | 1,981 |
|  | Actinic keratosis | 1,314 |
|  | Application site scab | 1,009 |
|  | Skin induration | 948 |
|  | Precancerous skin lesion | 943 |
|  | Injection site scab | 532 |
|  | Application site papules | 495 |
|  | Injection site macule | 213 |
| **Vascular Cutaneous** | Skin haemorrhage | 10,275 |
|  | Petechiae | 5,286 |
|  | Purpura | 3,810 |
|  | Ecchymosis | 2,921 |
|  | Cutaneous vasculitis | 2,329 |
|  | Haemorrhage subcutaneous | 1,944 |
|  | Immune thrombocytopenic purpura | 1,591 |
|  | Hypersensitivity vasculitis | 1,525 |
|  | Thrombotic thrombocytopenic purpura | 1,215 |
|  | Henoch-schonlein purpura | 1,084 |
|  | Livedo reticularis | 1,006 |
|  | Vascular purpura | 581 |
|  | Thrombocytopenic purpura | 492 |
|  | Telangiectasia | 481 |
|  | Vasculitic rash | 317 |
| **Wound Ulcer** | Wound | 19,123 |
|  | Skin ulcer | 14,316 |
|  | Wound infection | 6,739 |
|  | Postoperative wound infection | 4,262 |
|  | Decubitus ulcer | 4,037 |
|  | Pyoderma gangrenosum | 2,440 |
|  | Wound haemorrhage | 2,122 |
|  | Skin erosion | 2,117 |
|  | Wound complication | 2,014 |
|  | Wound secretion | 1,758 |
|  | Diabetic foot | 1,599 |
|  | Wound dehiscence | 1,382 |
|  | Skin wound | 1,153 |
|  | Infected skin ulcer | 962 |
|  | Diabetic foot infection | 802 |
| **Other Skin Changes** | Erythema | 130,784 |
|  | Dry skin | 104,017 |
|  | Acne | 60,846 |
|  | Skin burning sensation | 58,974 |
|  | Injection site erythema | 56,089 |
|  | Skin discolouration | 26,652 |
|  | Systemic lupus erythematosus | 25,279 |
|  | Pain of skin | 21,330 |
|  | Skin disorder | 20,470 |
|  | Skin fissures | 14,264 |
|  | Palmar-plantar erythrodysaesthesia syndrome | 14,050 |
|  | Application site erythema | 11,129 |
|  | Photosensitivity reaction | 8,877 |
|  | Skin reaction | 8,305 |
|  | Folliculitis | 7,876 |

# **Supplementary Table S6: Per-target × category disproportionality**

Reporting odds ratios (95% confidence intervals) for each BiTE molecular target against each Storgard category, computed within the disease-matched haematologic-oncology comparator cohort (n=1,062,195). Cells are formatted as ROR (95% CI). Cells with a 95% CI lower bound above 1 are described as nominal elevations and should be interpreted as exploratory descriptive findings rather than multiplicity-adjusted confirmatory signals. "n=0" or "n=1" indicates a cell count below the threshold for stable ROR estimation. Reference: non-BiTE haematologic-oncology drug exposure within the same cohort.

| **Category** | **CD19 (blinatumomab)** | **CD20 (4 agents)** | **BCMA (3 agents)** | **GPRC5D (talquetamab)** | **DLL3 (tarlatamab)** |
| --- | --- | --- | --- | --- | --- |
| **Bullous Dermatosis** | 0.07 (0.04–0.14) | 0.14 (0.08–0.26) | 0.09 (0.04–0.21) | 0.40 (0.21–0.77) | n=1 |
| **Eczematous** | 0.72 (0.46–1.12) | 0.42 (0.21–0.84) | 0.95 (0.57–1.59) | 1.26 (0.60–2.64) | n=0 |
| **Psoriasiform** | 0.29 (0.12–0.70) | 0.26 (0.08–0.80) | 0.83 (0.41–1.66) | n=0 | n=0 |
| **Rash** | 0.38 (0.33–0.45) | 0.38 (0.31–0.46) | 0.57 (0.48–0.67) | **1.49 (1.24–1.80)** | 0.31 (0.20–0.48) |
| **Severe Cutaneous Eruption** | 0.34 (0.15–0.76) | 0.33 (0.12–0.89) | 0.60 (0.27–1.34) | 0.56 (0.14–2.26) | n=1 |
| **Toxic Eruption** | 0.42 (0.20–0.88) | 0.62 (0.30–1.30) | **1.82 (1.13–2.94)** | n=0 | n=0 |
| **Urticaria Angioedema** | 0.11 (0.05–0.22) | 0.18 (0.09–0.34) | 0.26 (0.15–0.48) | 0.27 (0.10–0.72) | n=0 |
| **Hair Changes** | 0.13 (0.08–0.20) | 0.03 (0.01–0.10) | 0.03 (0.01–0.10) | **1.78 (1.33–2.38)** | n=1 |
| **Hyperhidrosis** | 0.25 (0.16–0.40) | 0.45 (0.30–0.68) | 0.45 (0.29–0.70) | **2.03 (1.41–2.92)** | 0.26 (0.09–0.82) |
| **Pruritus** | 0.10 (0.06–0.16) | 0.15 (0.09–0.24) | 0.27 (0.18–0.40) | 1.17 (0.85–1.62) | 0.32 (0.16–0.64) |
| **Skin Lesions** | 0.29 (0.15–0.56) | 0.24 (0.10–0.57) | 0.34 (0.15–0.76) | 1.13 (0.54–2.37) | n=0 |
| **Vascular Cutaneous** | 0.28 (0.15–0.52) | 0.08 (0.02–0.33) | 0.10 (0.02–0.39) | 0.56 (0.21–1.49) | n=1 |
| **Wound Ulcer** | 0.10 (0.06–0.18) | 0.08 (0.04–0.18) | n=1 | 0.09 (0.02–0.37) | n=0 |
| **Other Skin Changes** | 0.28 (0.23–0.34) | 0.24 (0.18–0.31) | 0.63 (0.52–0.75) | **5.65 (5.01–6.39)** | 0.16 (0.08–0.31) |

**Key findings from Table S6: (1) Talquetamab (GPRC5D) showed the clearest target-specific reporting pattern, with nominal elevations in "other skin changes" (ROR 5.65, encompassing nail and skin discolouration phenotypes), hyperhidrosis (ROR 2.03), hair changes (ROR 1.78), and rash (ROR 1.49). These patterns are compatible with tissues in which GPRC5D expression has been described, but they do not establish causality. (2) BCMA showed a single nominal elevation in toxic eruption (ROR 1.82), an exploratory finding warranting prospective verification. (3) Tarlatamab (DLL3) showed no individual category-level elevation, consistent with the small total event count; its early timing estimate in the main analysis was based on only eight reports with usable latency and should be considered descriptive and hypothesis-generating. (4) CD19 and CD20 BiTEs showed no category-level elevations.**

# **Canada Vigilance directional comparison**

The Canada Vigilance directional comparison used the same outcome and exposure dictionaries as the FAERS analysis where possible. Because Canada Vigilance lacks a reliable per-drug start date, analyses focused on reporting odds ratios and adjusted logistic regression rather than latency models. The Canadian dataset was considered supportive and hypothesis-generating rather than an independent validation.

**Supplementary Table S7: Canada Vigilance per-product report counts and cutaneous outcome counts**

| **Product** | **Reports, n** | **Any cutaneous AE, n** | **SCAR-broad, n** | **SJS/TEN, n** |
| --- | --- | --- | --- | --- |
| Blinatumomab (CD19) | 131 | 6 | 1 | 0 |
| Glofitamab (CD20) | 15 | 0 | 0 | 0 |
| Epcoritamab (CD20) | 65 | 4 | 0 | 0 |
| Mosunetuzumab (CD20) | 0 | 0 | 0 | 0 |
| Odronextamab (CD20) | 1 | 0 | 0 | 0 |
| Teclistamab (BCMA) | 270 | 18 | 0 | 0 |
| Elranatamab (BCMA) | 96 | 17 | 0 | 0 |
| Linvoseltamab (BCMA) | 1 | 0 | 0 | 0 |
| Talquetamab (GPRC5D) | 37 | 8 | 0 | 0 |
| Tarlatamab (DLL3) | 47 | 2 | 0 | 0 |

AE = adverse event; SCAR = severe cutaneous adverse reaction; SJS/TEN = Stevens-Johnson syndrome/toxic epidermal necrolysis. Counts are report counts, not incidence rates. Canada Vigilance included 662 unique BiTE-exposed reports; per-product and target-level counts may sum to 663 because one report listed more than one BiTE product or target.

**Supplementary Table S8: Canada Vigilance target-specific reporting odds ratios for any cutaneous adverse event**

| **Target** | **All-Canada ROR (95% CI)** | **Haem-onc ROR (95% CI)** |
| --- | --- | --- |
| CD19 | 0.33 (0.15-0.75) | 0.31 (0.14-0.70) |
| CD20 | 0.36 (0.13-0.98) | 0.33 (0.12-0.91) |
| BCMA | 0.73 (0.51-1.03) | 0.68 (0.48-0.96) |
| GPRC5D | 1.91 (0.87-4.17) | 1.77 (0.81-3.88) |
| DLL3 | 0.31 (0.07-1.27) | 0.29 (0.07-1.18) |

ROR = reporting odds ratio; CI = confidence interval. The haem-onc comparator was restricted to BiTE and non-BiTE haematologic-oncology drug reports.

## **Supplementary Figures:**

## **Supplementary Figure S1: Canada Vigilance BiTE association across two cutaneous tiers**

Reporting odds ratios with 95% confidence intervals for any cutaneous AE and SCAR-broad in all-Canada and disease-matched haematologic-oncology cohorts. Narrow SJS/TEN was not plotted because no BiTE-exposed reports were identified.

## **Supplementary Figure S2: Canada Vigilance 14-category cutaneous disproportionality panel**

Reporting odds ratios with 95% confidence intervals for BiTE-exposed versus non-BiTE reports across Storgard dermatological categories in all-Canada and disease-matched haematologic-oncology cohorts.

## **Supplementary Figure S3: Canada Vigilance target-specific cutaneous AE reporting pattern**

Target-level reporting odds ratios with 95% confidence intervals for any cutaneous AE, using the disease-matched haematologic-oncology comparator as reference. Canada Vigilance covered 2016Q1-2025Q4. These estimates provide a directional comparison only and should not be interpreted as independent validation.
